# Role of Toll-like receptors and interferon regulatory factors in different experimental heart failure models of diverse etiology: IRF7 as novel cardiovascular stress-inducible factor

**DOI:** 10.1371/journal.pone.0193844

**Published:** 2018-03-14

**Authors:** Peter Moritz Becher, Svenja Hinrichs, Nina Fluschnik, Jan K. Hennigs, Karin Klingel, Stefan Blankenberg, Dirk Westermann, Diana Lindner

**Affiliations:** 1 Department for General and Interventional Cardiology, University Heart Center Hamburg, Hamburg, Germany; 2 DZHK (German Center for Cardiovascular Research), partner site Hamburg/Kiel/Lübeck, Hamburg, Germany; 3 Section Pneumology, Department of Medicine II, University Medical Center Hamburg Eppendorf, Hamburg, Germany; 4 Cardiopathology, Institute for Pathology and Neuropathology, Eberhard-Karls-University Tübingen, Tübingen, Germany; University of Hong Kong, HONG KONG

## Abstract

Heart failure (HF) is a leading cause of morbidity and mortality in the western world. Although optimal medical care and treatment is widely available, the prognosis of patients with HF is still poor.

Toll-like receptors (TLRs) are important compartments of the innate immunity. Current studies have identified TLRs as critical mediators in cardiovascular diseases. In the present study, we investigated the involvement of TLRs and interferon (IFN) regulatory factors (IRFs) in different experimental HF models including viral myocarditis, myocardial ischemia, diabetes mellitus, and cardiac hypertrophy. In addition, we investigated for the first time comprehensive TLR and IRF gene and protein expression under basal conditions in murine and human cardiac tissue. We found that *Tlr4*, *Tlr9* and *Irf7* displayed highest gene expression under basal conditions, indicating their significant role in first-line defense in the murine and human heart. Moreover, induction of TLRs and IRFs clearly differs between the various experimental HF models of diverse etiology and the concomitant inflammatory status. In the HF model of acute viral-induced myocarditis, TLR and IRF activation displayed the uppermost gene expression in comparison to the remaining experimental HF models, indicating the highest amount of myocardial inflammation in myocarditis. In detail, *Irf7* displayed by far the highest gene expression during acute viral infection. Interestingly, post myocardial infarction TLR and IRF gene expression was almost exclusively increased in the infarct zone after myocardial ischemia (*Tlr2*, *Tlr3*, *Tlr6*, *Tlr7*, *Tlr9*, *Irf3*, *Irf7*). With one exception, *Irf3* showed a decreased gene expression in the remote zone post infarction. Finally, we identified *Irf7* as novel cardiovascular stress-inducible factor in the pathologically stressed heart. These findings on TLR and IRF function in the inflamed heart highlight the complexity of inflammatory immune response and raise more interesting questions for future investigation.

## Introduction

Heart failure (HF) is a leading cause of hospitalization and mortality in the western world [1]. Despite the implementation of optimal medical care and management of the HF syndrome [2, 3], prevalence, morbidity, mortality, and costs are still rising [4, 5]. The growth of our elderly population with increased prevalence of comorbidities such as coronary artery disease (CAD), myocardial infarction, hypertension, and diabetes that predispose those patients to this multifactorial syndrome is expected to rise HF prevalence in the future [6]. Current treatment strategies primarily slow the progression of the multifactorial HF syndrome. However, there is an important need to develop new preventative and restorable therapy options [2, 3]. In addition, the development of new HF therapies needs testing of the accepted therapeutic strategies in proper small and large animal models of advanced HF [7, 8]. The development of the complex HF syndrome involves several pathophysiological processes including activation of the immune system as trigger of left ventricular (LV) dysfunction and adverse cardiac remodeling [9].

Toll-like receptors (TLRs) are an essential compartment of the innate immune system and have been identified to induce gene expression of pro-inflammatory cytokines and chemokines [10]. Additionally, TLRs typically recognize viral and bacterial molecular patterns, respectively [10]. Moreover, necrotic cells and damaged extracellular matrix (ECM) release endogenous alarm signals named as “damage associated molecular patterns” (DAMPs) [11]. DAMPs utilize their sterile pro-inflammatory actions by stimulating members of the TLR family [11, 12]. However, TLRs and their signaling components are activated in experimental HF models and in the clinical setting of the HF syndrome [13–15]. There exist solid evidence indicating that TLRs play a significant role in the pathogenesis of coxsackievirus B3 (CVB3)-induced myocarditis, myocardial infarction, diabetic cardiomyopathy, and angiotensin II (AngII)-induced HF with LV dysfunction [16–22]. Furthermore, TLR gene expression is increased in cardiac tissue of patients suffering from severe HF [15, 23]. In addition, TLR gene expression pattern changes during the development of HF [11]. In the present study, we thus investigated comprehensive gene expression of TLRs and interferon (IFN) regulatory factors (IRFs) under basal conditions in murine and human cardiac tissue and in four well-established experimental HF models of diverse etiology and different inflammatory status.

## Material and methods

### Animals and ethics

All mice were bred under pathogen-free conditions in the animal facility of the University Medical Center Hamburg-Eppendorf. Moreover, all mice, 6–8 weeks of age, were kept on a C57BL/6 background and housed in the animal facility of the University Medical Center Hamburg-Eppendorf at 22°C with ad libitum access to water and standard laboratory chow diet (Lasvendi). Mice were housed in groups of up to four mice per cage with a 14h light and 10h dark cycle under SPF-conditions. All experiments were approved by the institutional board at the University Medical Center Hamburg-Eppendorf and Charité-Universitätsmedizin Berlin. In detail, surgery and animal care were provided following the *Guide for the Care and Use of Laboratory Animals* (National Institutes of Health, volume 25, no 28, revised 1996) and in accordance with federal regulations. The study protocols were approved by state authorities. Inhalative anesthesia with 2.5% vaporized isoflurane (Abbott, Germany) was used in all experiments (exclusive diabetic cardiomyopathy). Animals received analgesic drugs and kept under special care in the central animal laboratory of our institution. Monitoring of the animals included daily visits. Mice were sacrificed by cervical dislocation under isoflurane anesthesia.

### Heart failure models and study design

Four experimental HF failure models were used to analyze TLR and IRF activation under different inflammatory conditions. 1) In the model of CVB3-induced viral myocarditis, mice were randomly selected for inoculation with CVB3 (Nancy strain, 5×10^5^ plaque-forming units; i.p.) diluted in a final volume of 0.2 ml saline as previously described [16, 20, 24]. Cardiac tissue was collected 7 and 28 days after infection. 2) Myocardial infarction was induced by permanent occlusion of the left anterior descending artery (LAD occlusion) over a period of 5 days as previously explained [25]. For subsequent analyses LV tissue was separated in two different sections post infarction: remote zone and infarction zone (scar tissue) and analyzed separately. 3) Diabetes was induced by injection of streptozocin (STZ) (50 mg/kg i.p. for 5 days) over a period of 8 weeks, while the others served as nondiabetic controls. Hyperglycemia (glucose >22 mmol/l) was confirmed 7 days later using a reflectance meter (Acutrend; Boehringer, Mannheim, Germany), as well as at the end of the study (glucose > mmol/l) [26, 27]. 4) In AngII-induced HF, Ang II (2.4 mg/kg per day s.c.; Bachem, Weil am Rhein, Germany) or sterile ringer solution (Merck, Darmstadt, Germany) was continuously infused via osmotic pumps over a period of 3 weeks (model 2004, ALZET, Cupertino, CA) [28, 29]. Throughout all experiments, 6–8 mice were analyzed per group.

### Tissue preparation

After euthanization (time point depending on the different HF models), LV tissues were excised and immediately snap frozen in liquid nitrogen. For further analyses tissue was stored at -80°C.

### RNA isolation from cardiac tissue samples

Total RNA was isolated from frozen tissue sections using Trizol reagent. Disruption of the tissue occurred during 10 min of vigorous shaking, followed by extraction of RNA by adding chlorophorm. After mixing and centrifugation, the aqueous phase containing the RNA was collected. Isopropanol was added and the samples were centrifuged for 15 min at 4°C at high speed to precipitate the RNA. The obtained RNA pellet was further purified using RNeasy Mini Kit (Qiagen) following the manufacturer’s instructions. Determination of the nucleic acid concentration was performed by measuring the absorbance at 260 nm using a Nanodrop 2000c spectrophotometer. RNA was stored at -80°C for further analyses.

### Reverse transcription and relative gene expression analysis

Reverse transcription of RNA was carried out using the High Capacity Kit (Life Technologies, Germany). One μg total RNA isolated from cardiac tissue was reversely transcribed for 2 h at 37°C followed by an inactivation step for 5 min at 85°C. The resulting cDNA was diluted to a final working concentration of 10 ng/μL for tissue samples. To assess the mRNA expression of the target genes real time PCR was performed using 5 μl of the gene expression master mix (life technologies) and the 0.5 μl of the gene expression assay for *Tlr1* (Mm00446095_m1), *Tlr2* (Mm00442346_m1), *Tlr3* (Mm01207404_m1), *Tlr4* (Mm00445273_m1), *Tlr5* (Mm00546288_m1), *Tlr6* (Mm02529782_s1), *Tlr7* (Mm00446917_m1), *Tlr8* (Mm04209873_m1), *Tlr9* (mM00446193_m1), *Irf3* (Mm00516784_m1), and *Irf7* (Mm00516788_m1) (each includes forward and reverse primers as well the fluorescently FAM-labeled probe). As template 1 μl cDNA was used in a final volume of 10 μl, each performed in duplicates. As an endogenous control, the gene expression of 18S (Hs99999901_s1) was assessed. To calculate the absolute gene expression, data were normalized to the housekeeping gene by using the formula 2^‐ΔCt^ and plotted as absolute mRNA expression. Whereas the relative gene expression data were calculated further normalized using the formula using the formula 2^‐ΔΔCt^ and plotted as x-fold to untreated control as described previously [30].

### In silico protein expression analysis

A protein abundance database search (https://pax-db.org) was conducted for protein expression datasets from human cardiac tissue [31, 32].

An integrated dataset covering 51% of the human proteome with an Interaction Consistency Score of 22.72 was used. Normalized protein abundance data in parts per million (ppm) was extracted for proteins available in the dataset and plotted as dots for comparison.

### Statistical analysis

All statistical analyses were performed using Graph Pad Prism 7 software (GraphPad Software, La Jolla, CA). Statistical comparison of two groups was performed using the nonparametric Mann- Whitney *U* test with *p* values <0.05 considered statistically significant. Statistical comparison of more than two groups were compared using Kruskal-Wallis-test followed by Dunn’s posttest with P values < 0.05 considered statistically significant.

## Results

### Gene expression and protein profiling of the plasma membrane and intracellular localized TLRs and IFN regulatory factors under basal conditions in murine and human cardiac tissue

**Fig 1:**
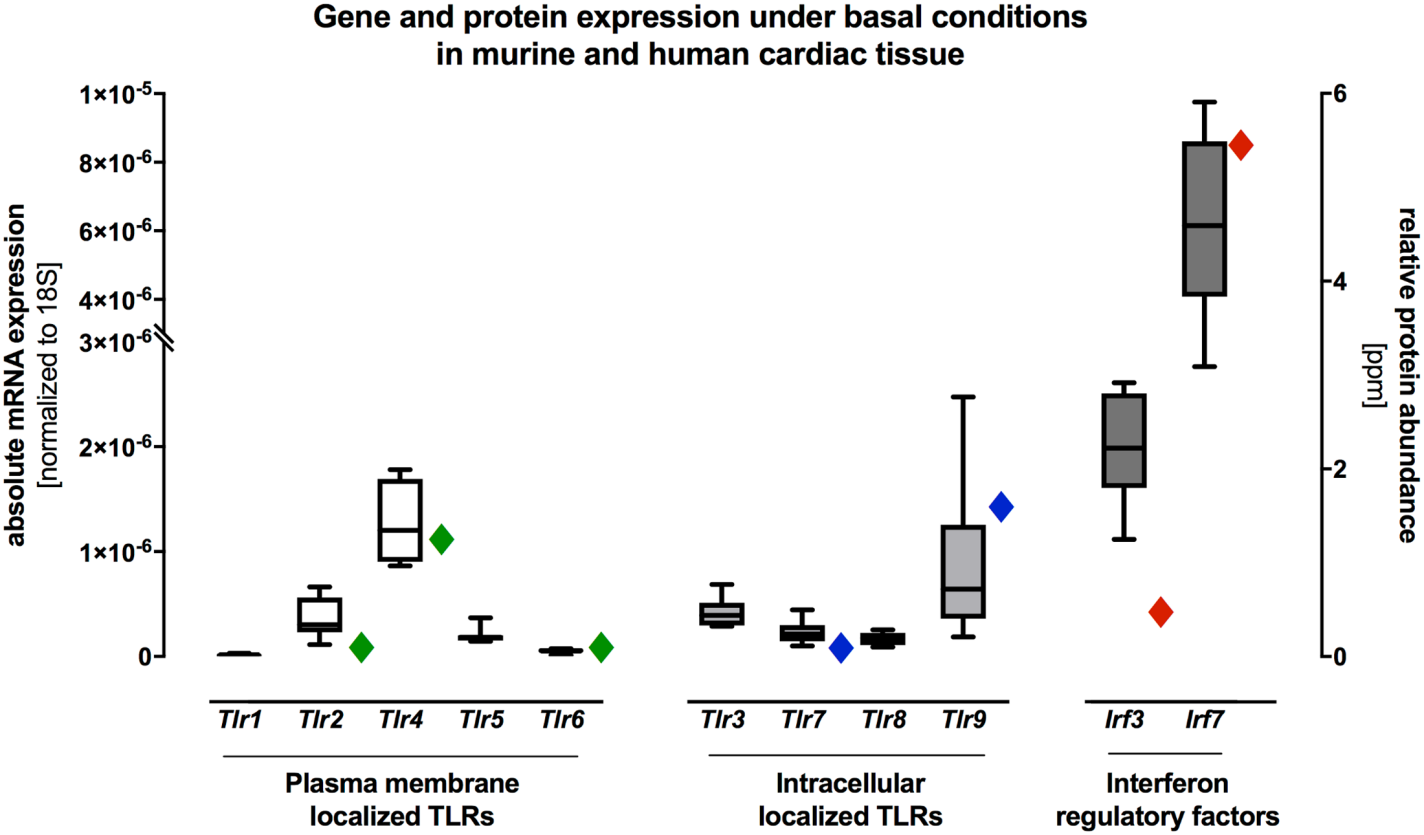
Gene and protein expression of plasma membrane and intracellular localized TLRs and of the IFN regulatory factors IRF3 and IRF7 in murine and human cardiac tissue under basal conditions. Cardiac tissue of healthy C57BL/6J wildtype mice was used for TaqMan based gene expression analysis of various TLRs and their IFN regulatory factors *Irf3* and *Irf7* under basal conditions. Gene expressions of *Tlr4*, *Tlr9*, *Irf3* and *Irf7* were highly expressed when compared to the remaining TLRs under basal conditions in murine cardiac tissue. The highest gene expression under basal condition was detected for *Irf7*. In addition, we detected similar human protein expression patterns when compared with murine gene expression in cardiac tissue under basal conditions. Data are presented in box plots as absolute mRNA expression normalized to the house keeping gene *Cdkn1b* and human protein abundance are presented as rhombus sign on the right-hand of each mRNA expression.

Gene and protein expression of the plasma membrane localized *Tlr1*, *Tlr2*, *Tlr4*, *Tlr5*, *Tlr6* the intracellular localized *Tlr3*, *Tlr7*, *Tlr8*, *Tlr9*, and the IFN regulatory factors *Irf3* and *Irf7* were systematically analyzed under basal conditions in murine and human cardiac tissue (Fig 1). As shown in Fig 1, mRNA expression of the plasma membrane localized *Tlr4* and the intracellular localized *Tlr9* displayed the highest gene expression levels under basal conditions in murine cardiac tissue. Moreover, we analyzed the mRNA expression levels of the IFN regulatory factors *Irf3* and *Irf7*. In detail, we generally detected an increased mRNA expression of *Irf3 and Irf7* when compared to the remaining TLRs in murine cardiac tissue under basal conditions. Remarkably, *Irf7* displayed by far the highest gene expression under healthy conditions in the murine heart (Fig 1). In addition, absolute protein abundance levels were conducted for protein expression datasets from human cardiac tissue. The relative protein abundance showed also the highest protein expression of the plasma membrane localized TLR4 and the intracellular localized TLR9 (Fig 1). Additionally, the IFN regulatory factor IRF7 displayed the highest protein expression in the human heart under basal conditions (Fig 1).

### Gene expression profiling of the plasma membrane localized TLRs in HF models of viral myocarditis, myocardial infarction, diabetes mellitus, and cardiac hypertrophy

Gene expression of the plasma membrane localized *Tlr1*, *Tlr2*, *Tlr4*, *Tlr5*, and *Tlr6* was systematically analyzed in hearts from diseased and healthy C57BL/6J mice (four different HF models).

**Fig 2:**
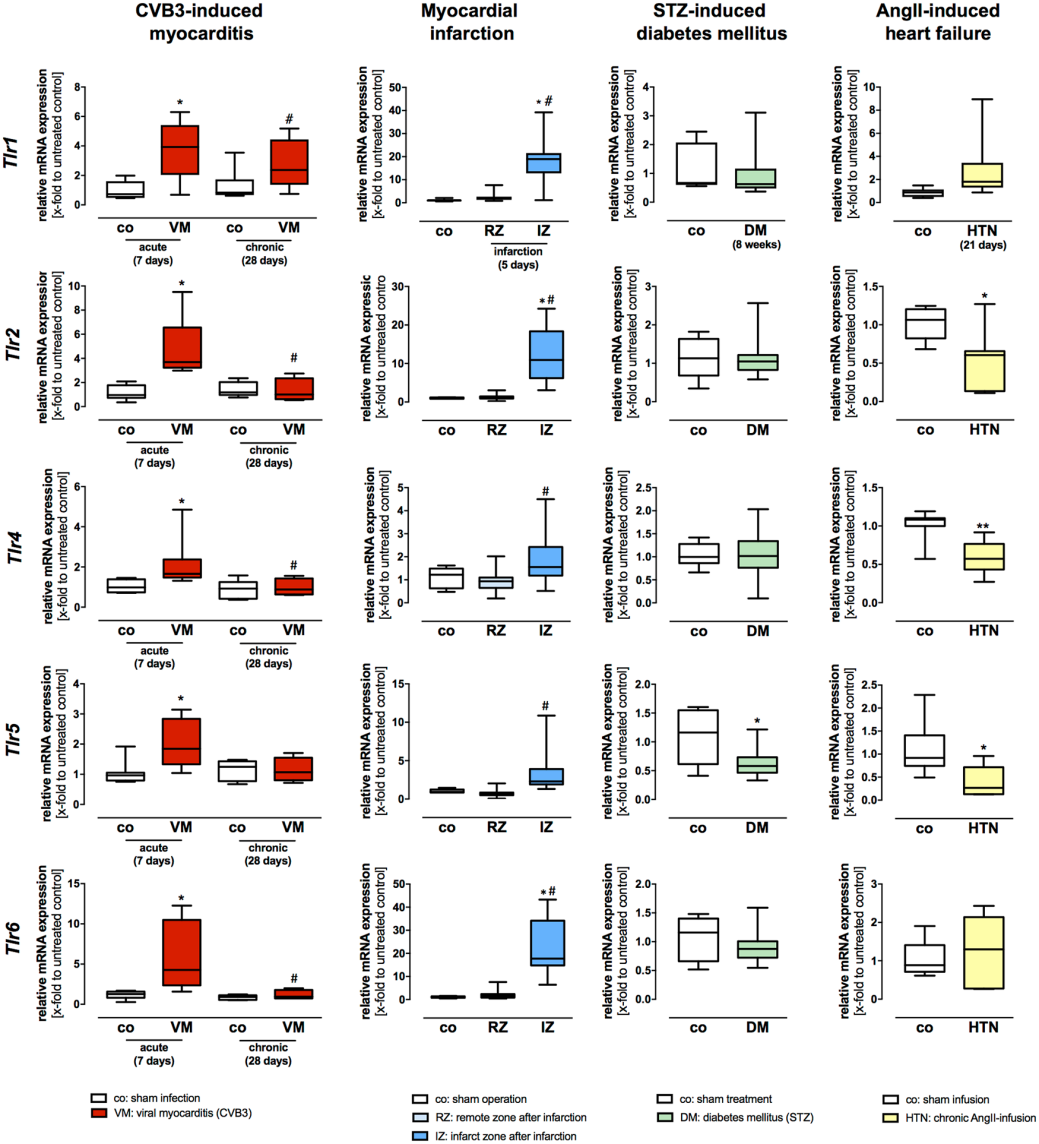
Gene expression levels of the plasma membrane localized TLRs *Tlr1*, *Tlr2*, *Tlr4*, *Tlr5*, and *Tlr6* in different heart failure models of diverse etiology. Cardiac tissue of healthy and diseased C57BL/6J mice was used for TaqMan based gene expression analysis of the plasma membrane localized TLRs *Tlr1*, *Tlr2*, *Tlr4*, *Tlr5*, and *Tlr6*. Expression levels of cardiac tissue from control mice are shown as white boxes, from diseased animals as red, blue, green or yellow boxes corresponding to the analyzed heart failure model. In viral-induced myocarditis (shown in red), gene expression of *Tlr1*, *Tlr2*, *Tlr4*, *Tlr5*, and *Tlr6* was highly increased 7 days after infection compared to healthy controls. However, *Tlr2* and *Tlr6* displayed the highest increase during acute myocarditis. The initially increased gene expression levels returned almost with exception to basal levels 28 days after infection. In the model of myocardial infarction (shown in blue), gene expression of *Tlr1*, *Tlr2*, *Tlr4*, *Tlr5*, and *Tlr6* was highly increased 5 days post infarction in the infarction zone when compared to the remote zone. In the remote zone, no increased TLR gene expression was observed. In STZ-induced diabetic cardiomyopathy (shown green), a decreased gene expression of *Tlr5* in comparison to their healthy controls was detected; the remaining plasma membrane localized TLRs displayed no changes in gene expression levels. In the heart failure model caused by chronic AngII-infusion for 21 days (shown in yellow), all plasma membrane localized TLRs displayed a significant decrease when compared to their controls. Data are presented in box plots as relative mRNA expression in fold change to the corresponding untreated control using the formula 2^−ΔΔCt^. * = significantly different compared to corresponding control; ^#^ = significantly different compared to VM (acute—7 days) or RZ (remote zone).

1) Myocarditis. In acute CVB3 myocarditis (7 days post infection (pi)) all plasma membrane localized TLRs showed an increased gene expression, however, the highest increase in gene expression levels displayed *Tlr2* and *Tlr6* (Fig 2). At the chronic stages of myocarditis (28 days pi CVB3) only *Tlr2*, *Tlr4* and *Tlr6* showed still an increased gene expression (Fig 2). 2) Myocardial infarction. Two different zones of the LV after infarction: the remote zone (non-infarcted myocardium) and the infarction zone (infarcted scar area) were analyzed. After myocardial infarction, gene expression levels of the plasma membrane localized *Tlr2* and *Tlr6* were significantly increased when compared to their healthy controls (Fig 2). Moreover, gene expression of *Tlr1*, *Tlr2*, *Tlr4*, *Tlr5*, and *Tlr6* was significantly increased 5 days post infarction in the infarction zone when compared to the remote zone (Fig 2). However, *Tlr2* and *Tlr6* displayed the highest increase in mRNA expression in the infarction zone when compared with the remaining plasma membrane localized TLRs (Fig 2). 3) Diabetic cardiomyopathy. There exists evidence that increased glucose levels induce TLR expression in human monocytes [33]. Therefore, we examined TLR gene expression levels in diabetic cardiomyopathy. Gene expression of the plasma membrane localized *Tlr5* was significantly decreased compared to their healthy controls 8 weeks after STZ-induced diabetes (Fig 2). However, the remaining plasma membrane localized TLRs displayed no significant differences in gene expression in diabetic cardiomyopathy (Fig 2). 4) AngII-mediated cardiac hypertrophy. Recently, it has been shown that TLRs are significantly involved in the development of AngII-induced cardiac hypertrophy and cardiac dysfunction [34]. Thus, we examined gene expression levels of TLRs in HF caused by chronic AngII-infusion for 21 days. Gene expression of the plasma membrane localized *Tlr2*, *Tlr4*, and *Tlr5* was significantly decreased compared to their controls 21 days after AngII-induced HF (Fig 2).

### Gene expression profiling of the intracellular localized TLRs in HF models of viral myocarditis, myocardial infarction, diabetes mellitus, and cardiac hypertrophy

Gene expression of the intracellular localized *Tlr3*, *Tlr7*, *Tlr8*, and *Tlr9* was systematically analyzed in hearts from diseased C57BL/6J and C57BL/6J wildtype mice (four different HF models).

**Fig 3:**
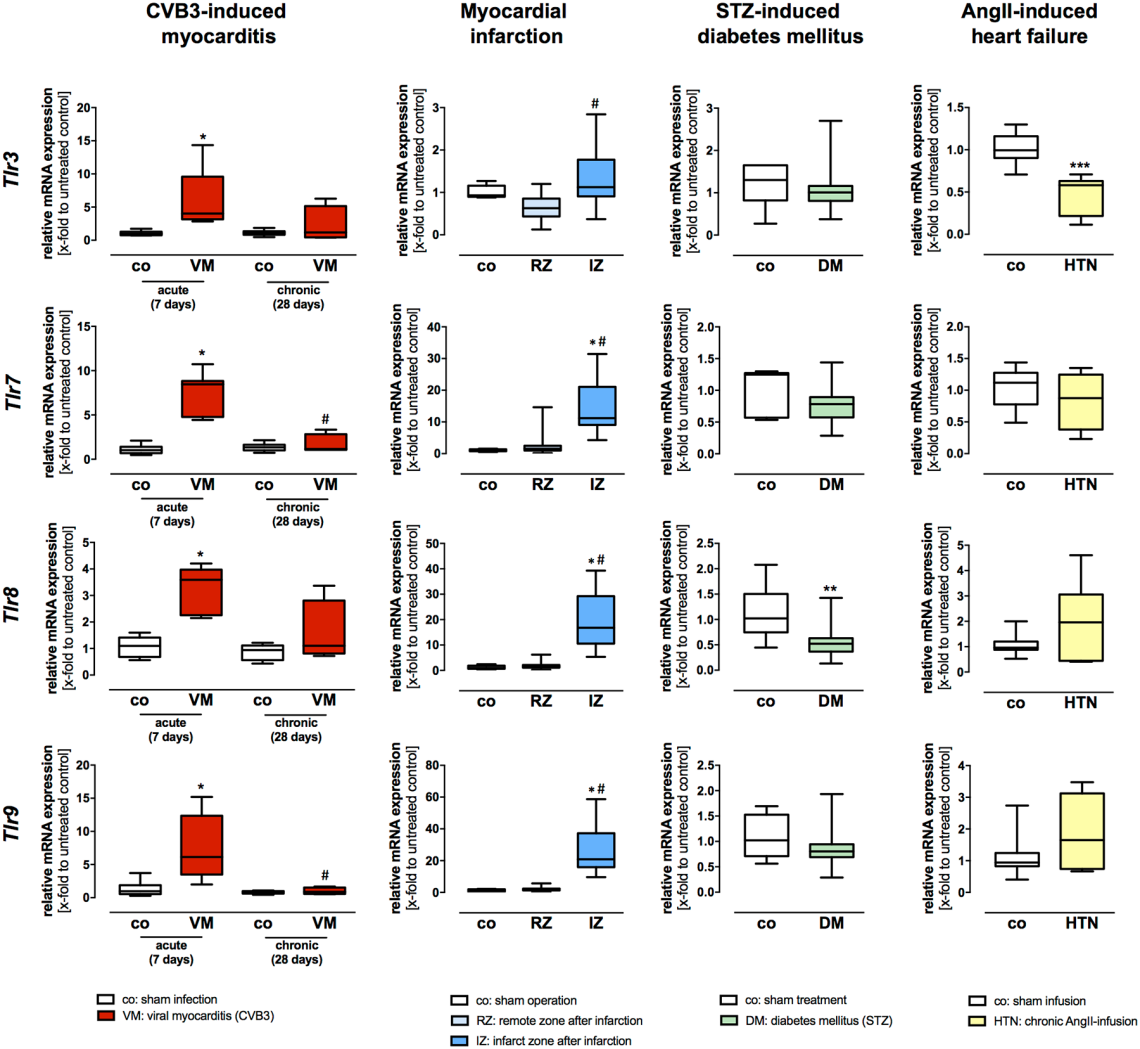
Gene expression levels of the intracellular localized TLRs *Tlr3*, *Tlr7*, *Tlr8*, and *Tlr9* in different HF models of diverse etiology. Cardiac tissue of healthy and diseased C57BL/6J mice was used for TaqMan based gene expression analysis of the plasma membrane localized TLRs *Tlr3*, *Tlr7*, *Tlr8*, and *Tlr9*. Expression levels of cardiac tissue from control mice are shown as white boxes, from diseased animals as red, blue, green, or yellow boxes corresponding to the analyzed heart failure model. In viral-induced myocarditis (shown in red), gene expression of *Tlr3*, *Tlr7*, *Tlr8*, and *Tlr9* was highly increased 7 days after infection compared to healthy controls. However, *Tlr3*, *Tlr7* and *Tlr9* displayed the highest increase during acute myocarditis and the initially increased gene expression levels returned almost with exception to basal levels 28 days after infection. Moreover, in the model of myocardial infarction (shown in blue), gene expression of *Tlr3*, *Tlr7*, *Tlr8*, and *Tlr9* was highly increased 5 days post infarction in the infarct zone when compared to the remote zone. In addition, the TLRs *Tlr7*, *Tlr8*, and *Tlr9* showed the highest increase in gene expression levels after myocardial ischemia. In the remote zone, no increased TLR gene expression was observed. In STZ-induced diabetic cardiomyopathy (shown in green), a decreased gene expression of *Tlr8* in comparison to their healthy controls was detected; the remaining TLRs displayed no changes in gene expression levels. In the heart failure model caused by chronic AngII-infusion for 21 days (shown in yellow) only the intracellular localized *Tlr3* displayed a significant decrease when compared to their controls. Data are presented as relative mRNA expression in fold change to the corresponding untreated control using the formula 2^−ΔΔCt^. * = significantly different compared to corresponding control; ^#^ = significantly different compared to VM (acute—7 days) or RZ (remote zone).

1) Myocarditis. In acute CVB3 myocarditis (7 days post infection (pi) with CVB3) all intracellular localized TLRs showed an increased gene expression (Fig 3). Highest gene expression levels displayed *Tlr3*, *Tlr7*, and *Tlr9* 7 days pi CVB3 with a persistently increased gene expression at the chronic stage of disease (28 days pi) when compared to their healthy controls (Fig 3). However, the intracellular localized TLRs *Tlr3*, *Tlr7*, *Tlr8*, and *Tlr9*, responsible for the recognition of viral nucleic acids, displayed generally higher gene expression levels when compared to the plasma membrane localized TLRs (Fig 3). 2) Myocardial infarction. Two different zones of the LV after infarction: the remote zone (non-infarcted myocardium) and the infarction zone (infarct scar area) were analyzed. After myocardial infarction, gene expression levels of the intracellular localized *Tlr7*, *Tlr8*, and *Tlr9* were significantly increased when compared to their healthy controls (Fig 3). Moreover, gene expression of *Tlr3*, *Tlr7*, *Tlr8*, and *Tlr9* was significantly increased 5 days post infarction in the infarction zone when compared to the remote zone (Fig 3). However, the intracellular localized *Tlr7*, *Tlr8*, and *Tlr9* displayed the highest increase in mRNA expression in the infarction zone when compared with the remaining TLRs (Fig 3). 3) Diabetic cardiomyopathy: Gene expression of the intracellular localized *Tlr8* was significantly decreased compared to their healthy controls 8 weeks after STZ-induced diabetes (Fig 3). However, the remaining TLRs displayed no significant differences in gene expression during diabetic cardiomyopathy. 4) AngII-mediated cardiac hypertrophy. Gene expression of the intracellular localized *Tlr3* was significantly decreased compared to their sham controls 21 days after AngII-induced HF (Fig 3).

### Gene expression profiling of the IFN regulatory factors IRF3 and IRF7 in HF models of viral myocarditis, myocardial infarction, diabetes mellitus, and cardiac hypertrophy

**Fig 4:**
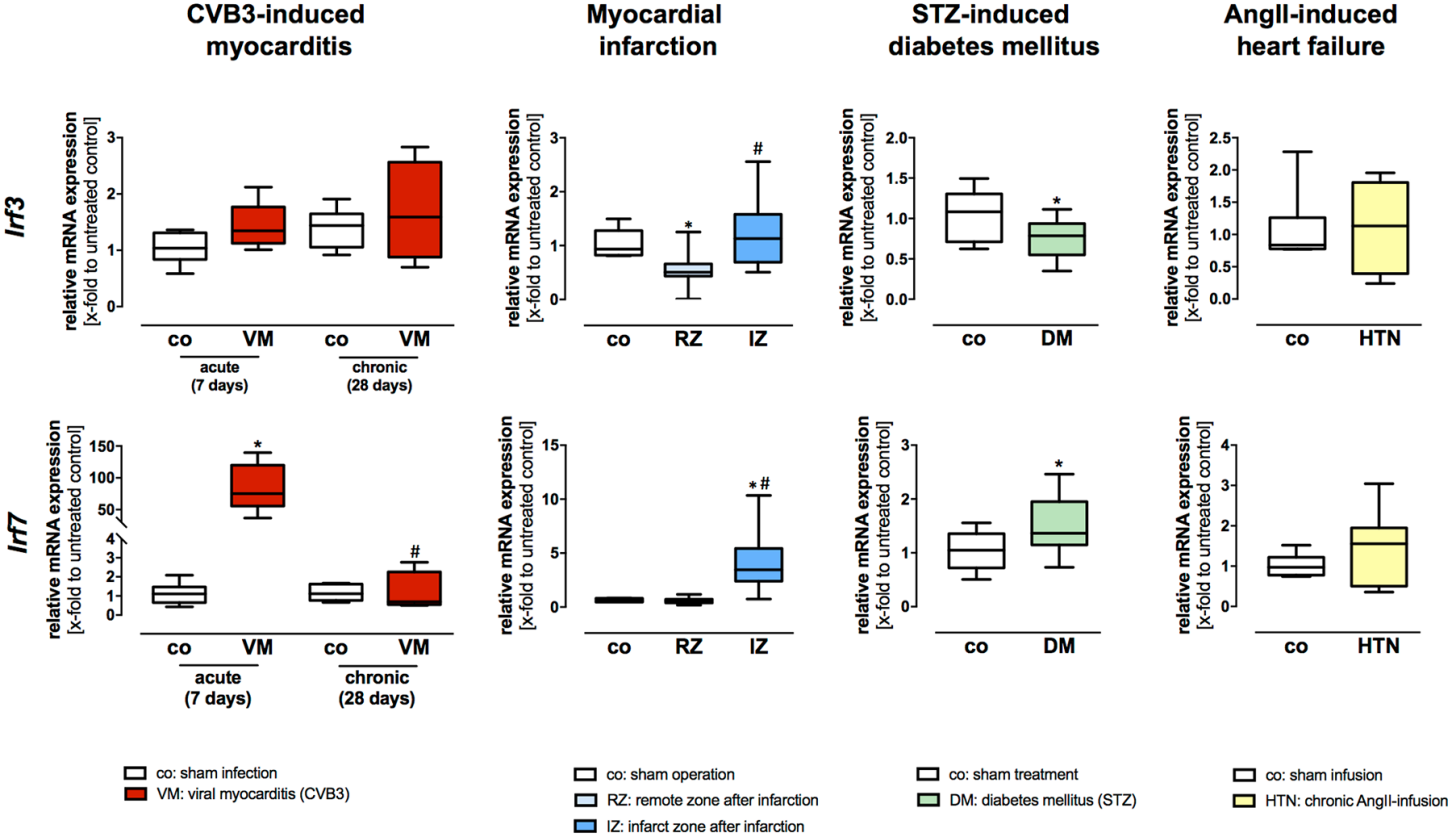
Gene expression levels of the IFN regulatory factors *Irf3* and *Irf7* in different HF models of diverse etiology. Cardiac tissue of healthy and diseased C57BL/6J mice was used for TaqMan based gene expression analysis of the IFN regulatory factors *Irf3* and *Irf7*. Expression levels of cardiac tissue from control mice are shown as white boxes, from diseased animals as red, blue, green or yellow boxes corresponding to the analyzed heart failure model. In viral-induced myocarditis (shown in red), gene expression of *Irf7* showed by far the highest increase 7 days after infection compared to healthy controls. However, *Irf3* displayed no changes in gene expression during viral myocarditis. Moreover, in the model of myocardial infarction (shown in blue), gene expression of *Irf3* and *Irf7* was highly increased 5 days post infarction in the scar tissue when compared to the non-infarcted LV or sham, whereas *Irf7* showed a clearly higher gene expression when compared to the expression of *Irf3*. In STZ-induced diabetic cardiomyopathy (shown in green), an opposite effect on gene expression of *Irf3* and *Irf7* was observed. Whereas, *Irf3* showed a decreased gene expression, *Irf7* displayed an increased gene expression under diabetic conditions. However, in the heart failure model caused by chronic AngII-infusion for 21 days (shown in yellow), no differences in gene expression of *Irf3* and *Irf7* were detected. Data are presented in box plots as relative mRNA expression in fold change to the corresponding untreated control using the formula 2^−ΔΔCt^. * = significantly different compared to corresponding control; ^#^ = significantly different compared to VM (acute—7 days) or RZ (remote zone).

1) Myocarditis. *Irf3* showed no significant differences in gene expression at the acute stadium after CVB3 infection (Fig 4). Interestingly, *Irf7* showed by far the highest increase in gene expression 7 days post infection in comparison to the remaining plasma membrane and intracellular localized TLRs (Fig 4). In addition, *Irf7* showed an increased gene expression at the chronic disease stadium 28 days after viral infection when compared to their healthy controls (Fig 4). 2) Myocardial infarction. Two different zones of the LV after infarction: the remote zone (non-infarcted myocardium) and the infarction zone (infarct scar area) were analyzed. The IFN regulatory factors *Irf3* and *Irf7* showed an increased gene expression 5 days after infarction in the infarction zone (Fig 4). Interestingly, *Irf3* displayed a decreased mRNA expression in the remote zone when compared to sham 5 days post infarction (Fig 4). 3) Diabetic cardiomyopathy. *Irf3* showed a significantly reduced gene expression during STZ-induced diabetes (Fig 4). In contrast to these findings, *Irf7* showed a significantly increased gene expression when compared to sham animals (Fig 4). 4) AngII-mediated cardiac hypertrophy. *Irf3* and *Irf7* displayed no significant differences in gene expression 21 days after AngII-induced HF in comparison to their controls (Fig 4).

## Discussion

We found that 1) *Tlr4*, *Tlr9* and the IFN regulator factors *Irf3* and *Irf7* showed the highest expression levels under basal conditions in the murine and human heart, 2) regulation of TLR and IRF gene expression differs between the various experimental HF models of diverse etiology and the concomitant inflammatory status, 3) TLR and IRF gene expression is preferentially increased in the infarction zone after myocardial ischemia, whereas no TLR activation was detected in the remote zone, and 4) *Irf7* might act as cardiovascular stress-inducible factors in the pathologically stressed heart.

### TLR and IRF gene profiling in murine and human cardiac tissue under basal conditions

Growing evidence indicates that TLRs and their activation can act as stress sensors in response to sterile tissue injury. TLRs recognize various endogenous danger molecules by DAMP recognition [12, 35]. TLR1, TLR2, TLR4, TLR5, and TLR6 are predominantly expressed on the cell surface and responsible for the recognition of microbial components (lipids, lipoproteins, proteins) [12]. In contrast to the above-mentioned TLRs, TLR3, TLR7, TLR8, and TLR9 are endosomal localized and responsible for the detection of microbial nucleic acids such as double-stranded RNA by TLR3, single-stranded RNA by TLR7 and TLR8, and the DNA by TLR9 [12, 36]. TLRs are ubiquitously expressed. However, the highest TLR expressions were detected in inflammatory immune cells. Nevertheless, TLRs are also expressed in cardiac myocytes, fibroblasts, and vascular smooth muscle cells [12, 15]. However, basal gene expression of TLRs under physiological condition is largely unexplored. In the present study, we determined that *Tlr4* and *Tlr9* showed the highest gene expression levels in the healthy murine heart. These findings could increase the relevance of *Tlr4* and *Tlr9* as highly involved TLRs in first-line defense against exogenous pathogens and receptor of sterile inflammatory processes. Furthermore, TLR expression patterns changes in advanced HF. Moreover, in sterile and non-infected human and murine heart tissue the TLR4 expression is primarily observed in cardiac myocytes [15]. In the heart from HF patients, focal areas of increased TLR4 expression were observed [23]. However, the causality of the different TLR4 gene expression has yet not been identified [23]. These findings suggest that TLRs and their downstream signaling are significantly involved in inflammatory conditions in advanced HF.

Interestingly, the IFN regulators factors *Irf3* and especially *Irf7* also showed high mRNA expression under basal conditions. Current experimental and clinical studies identified important functions of the IRFs in cardiovascular diseases and the HF syndrome [37].

Remarkably, we found similar human protein expression patterns when compared with murine gene expression in cardiac tissue under basal conditions. In detail, relative protein abundance under basal conditions in human cardiac tissue showed the highest protein expression of the plasma membrane localized TLR4 and the intracellular localized TLR9 (Fig 1). Additionally, the IFN regulatory factor IRF7 displayed the highest protein expression in the human heart under basal conditions (Fig 1). In our study, TLR and IRFs expression analysis showed a large overlapping in gene and protein expression levels under basal conditions in the murine and human cardiac tissue.

### Intense TLR and IRF induction in viral myocarditis

Myocarditis can be caused by multiple exogenous factors, such as viruses, bacteria, drugs, and as well as endogenous stimuli [38, 39]. In this regard, the CVB3 is cytopathic for cardiac cells in humans and mice [40]. However, invading pathogen-associated molecular patterns (PAMPs) are rapidly recognized by the innate immune response. The recognition of so named PAMPs occurs by pattern recognition receptors (PRRs), such as TLRs [12, 41]. In the present study, comprehensive induction of TLR gene expression during the acute and chronic stage of enteroviral myocarditis was also observed. During acute CVB3-induced myocarditis most of the plasma membrane and intracellular localized TLRs showed an enhanced gene expression, however, *Tlr2*, *Tlr3*, *Tlr6*, *Tlr7*, and *Tlr9* displayed by far the highest increase of mRNA expression during acute disease. Interestingly, merely the intracellular localized TLRs *Tlr3*, *Tlr8*, and *Tlr9* demonstrated elevated gene expression also at the chronic stage of myocarditis (28 days pi CVB3). Moreover, it has been investigated by others that the *Tlr8* has a significant prognostic impact in patients suffering from enterovirus-associated cardiomyopathy [42]. These findings suggest that preferentially the intracellular localized *Tlr3*, *Tlr8*, and *Tlr9*, which are mainly responsible for viral recognition, are significantly involved in the antiviral immune response also at chronic stages after CVB3 infection. Importantly, the antiviral IFN-ß expression is controlled by TLR downstream signaling. Particularly, the intracellular localized TLR3 is responsible for the recognition of dsRNA [43]. Moreover, the deficiency of TLR3 led to an aggravation of acute myocarditis and an increased mortality in mice [43]. In addition, the lack of the TIR-domain-containing adapter-inducing interferon-ß (TRIF) resulted in an overwhelming cardiac immune response and an impaired survival after acute CVB3-induced viral myocarditis in mice [16]. In this regard, highest gene expression by far (83-fold increased expression to controls) displayed the IFN regulatory factor *Irf7* during acute CVB3 myocarditis. In contrast to these findings, *Irf3* displayed no significant changes during acute or chronic myocarditis. Importantly, *Irf7* is a transcription factor responsible for the amplification of the IFN response in viral myocarditis. While the initial IFN-ß production depends preferentially on IRF3 induction [44, 45], the robust IFN-ß production depends on IRF7 [46]. These findings could improve our understanding of IRF7 gene expression and additional downstream IFN pathway induction for the host defense to CVB3-induced myocarditis. In addition, Omura and colleagues identified the induction of IRF7 as an inflammatory marker of the innate immune system to detect the acute phase of myocarditis and inflammatory cardiomyopathy [47]. In line with these findings, IRFs could act as new cardiovascular stress-inducible factors for inflammatory cardiomyopathy [48]. Our results underline the potential disease-phase dependent role of IRF7 to robust IFN induction in acute viral myocarditis.

### TLR and IRF induction preferentially in the infarction zone after myocardial infarction

Tissue injury produces endogenous stimuli leading to the activation of the innate immune system. These endogenous molecules are part of the large family of inflammatory immune mediators are known as DAMPs [49]. However, necrotic cardiac myocytes due to myocardial ischemia release endogenous DAMPs, which is associated with a significant induction of TLR4 expression [12]. The induction of TLR4 leads to increased expression of pro-inflammatory cytokines and chemokines, which cause intense inflammatory immune responses and additional injury to previously injured cardiac tissue. Interestingly, the activation of TLR4 signaling pathways showed a correlation with infarct severity but not with the level of myocardial inflammation [12, 50]. In addition, platelet activation can occur after acute myocardial infarction and despite treatment with anti-platelet therapy. TLRs may represent an alternative platelet activation pathway. In this context, it has been shown that the platelet-TLR2/1 activation pathway is functional post-AMI and despite treatment with anti-platelet therapy [51]. However, DAMPs can induce sterile inflammatory immune response after acute coronary artery occlusion [7]. DAMPs such as S100 proteins and the heat-shock proteins have been detected to be locally released after acute myocardial infarction [52]. Recognition of these DAMPs by TLRs (e.g. TLR3 and TLR4) can trigger the induction of additional inflammatory downstream pathways [11, 12, 53, 54]. Our results offer an increased gene expression of all TLRs in the infarction zone when compared to the remote zone 5 days post infarction. However, *Tlr1*, *Tlr2*, *Tlr6*, *Tlr7*, *Tlr8*, and *Tlr9* displayed highly increased mRNA expression in the infarcted area when compared with the remaining TLRs. Moreover, *Irf3* and *Irf7* showed also an increased gene expression 5 days after infarction in the scar tissue. However, TLR2 knockout mice displayed an improved LV function accompanied with a reduced infarct area due to a reduced TLR2-mediated leucocyte influx. Similar to TLR2, a reduction of TLR4-mediated signaling in mice showed a significant infarct size reduction and improved cardiac remodeling [20,24–29]. In this study, we observed highly increased gene expression levels of *Tlr1*, *Tlr2*, *Tlr6*, *Tlr7*, *Tlr8*, *Tlr9* as well as *Irf7* in the scar tissue after myocardial infarction, indicating their relevance to a proper cardiac wound healing. Interestingly, we observed a decreased gene expression of *Irf3* in the non-infarcted remote zone. These observations underline the hypothesis that IRF3 could be significantly involved in cardiac remodeling and fibrosis [55].

### Inverse gene expression of TLRs and IRFs in diabetic cardiomyopathy

Experimental studies investigated the role of TLRs in cardiac inflammation during diabetes mellitus (DM) type I and II [56]. Interestingly, increased TLR2 and TLR4 gene expression levels were associated with pathological increased blood glucose levels [57, 58]. Current studies displayed increased TLR expressions, particularly TLR2 and TLR4, in the setting of diabetic conditions [12, 59, 60]. Additionally, a large number of endogenous signaling molecules may be potent activators of the innate immune response by induction of the TLRs releasing of pro-inflammatory cytokines and chemokines from inflammatory immune cells. In DM type I, there only exist very limited data on the levels of endogenous ligands of the innate immune system [61–63]. Recently, in DM type II endogenous and sterile ligands of TLR2 and TLR4 have been identified and could act as DAMPs under diabetic conditions [11]. Interestingly and in contrast to these findings, our results showed a reduced gene expression of *Tlr5* and *Tlr8* 8 weeks after STZ-induced diabetes in the heart. In addition, the remaining TLRs displayed no significant differences in gene expression under diabetic conditions. *Irf3* showed a significantly reduced gene expression during STZ-induced diabetes. Contrarily, *Irf7* had a significantly increase gene expression when compared to sham animals. Moreover, a current study displayed a significant function for IRF7 in insulin sensitivity and energy metabolism [64]. These findings also suggest that IRF7 is involved in the etiology of metabolic abnormalities, which suggests a new strategy for treating DM type II and its consecutive complications [64].

### TLR and IRF activation in cardiac hypertrophy induced by chronic AngII-infusion

Hypertensive adverse cardiac remodeling, cardiac inflammation, and myocardial fibrosis, are known to be leading causes of chronic HF and LV hypertrophy [7, 65]. It has been shown in *in vivo* studies that the chronic endogenous exposure to AngII leads to LV dysfunction and hypertrophy [7, 28, 29]. Moreover, chronic infusion of AngII also led to contractile dysfunction in rodents [66]. Furthermore, AngII treatment increased TLR expression and enlarged pro-inflammatory cytokine expression in smooth muscle cells [67]. These findings suggest that AngII has a critical role in the development of LV dysfunction and hypertrophy. Interestingly, after chronic AngII-infusion we detected a down regulation of the TLRs *Tlr2*, *Tlr3*, *Tlr4*, and *Tlr5* in murine cardiac tissue. The expressions of the remaining TLRs and IRFs have been unaffected in this HF setting. These findings demonstrate the critical role of TLRs for the development of cardiac fibrosis in hypertensive heart disease associated with cardiac hypertrophy and LV dysfunction.

The experimental HF models of AngII-induced hypertrophy and diabetes are associated with lower level of myocardial inflammation and cell necrosis when compared to the acute HF models of MI and viral-induced myocarditis. However, the HF models of AngII-induced hypertrophy and diabetes display more pro-fibrotic than intense inflammatory effects. In addition, in this study the model of viral myocarditis is the only model with a direct pathogen interaction leading to an enormous inflammatory immune response. Moreover, there exists evidence that TLRs could also act as “stress sensors” in sterile inflammatory tissue injury. Endogenous ligands such as intracellular molecules and fragments of the extracellular matrix are significantly involved in TLR signaling and the resulting inflammatory immune response after myocardial damage. In this study, the diverse expression levels of TLRs and IRFs could be explained by the HF model associated grade of cardiac inflammation and the DAMP-activated TLR signaling.

## Conclusions

This study provides for the first time a systematical gene expression profiling of TLRs and IFN stimulating genes in murine and human cardiac tissue under basal conditions and in four different experimental HF models of diverse etiology. Our data determined a disease-phase dependent induction of TLRs and IRFs in different HF types and especially in the infarct and remote zone after acute myocardial ischemia, indicating their emerging role in intense inflammatory processes and a proper LV remodeling. In this context, *Irf7* might act as novel cardiovascular stress-inducible factor in the pathologically stressed heart. Although knowledge of the pathophysiology of the innate immune system in HF has substantially increased in the last years, future investigations, especially clinical trials are required to improve the effective treatment of patients by modulating the innate immune system.
